# Nano- and Micro-Porous Chitosan Membranes for Human Epidermal Stratification and Differentiation

**DOI:** 10.3390/membranes11060394

**Published:** 2021-05-27

**Authors:** Simona Salerno, Maria Penelope De Santo, Enrico Drioli, Loredana De Bartolo

**Affiliations:** 1 Institute on Membrane Technology, National Research Council of Italy (CNR-ITM), Via P. Bucci, cubo 17/C, 87036 Rende, Italy; e.drioli@itm.cnr.it; 2 Department of Physics and CNR-Nanotec, University of Calabria, Via P. Bucci, cubo 31/C, 87036 Rende, Italy; maria.desanto@fis.unical.it; 3 College of Chemical Engineering, Nanjing Tech University, Xinmofan Road, Nanjing 210009, China

**Keywords:** membranes, chitosan, nanoporous, microporous, epidermis, keratinocytes, differentiation, cell-material interactions

## Abstract

The creation of partial or complete human epidermis represents a critical aspect and the major challenge of skin tissue engineering. This work was aimed at investigating the effect of nano- and micro-structured CHT membranes on human keratinocyte stratification and differentiation. To this end, nanoporous and microporous membranes of chitosan (CHT) were prepared by phase inversion technique tailoring the operational parameters in order to obtain nano- and micro-structured flat membranes with specific surface properties. Microporous structures with different mean pore diameters were created by adding and dissolving, in the polymeric solution, polyethylene glycol (PEG Mw 10,000 Da) as porogen, with a different CHT/PEG ratio. The developed membranes were characterized and assessed for epidermal construction by culturing human keratinocytes on them for up to 21 days. The overall results demonstrated that the membrane surface properties strongly affect the stratification and terminal differentiation of human keratinocytes. In particular, human keratinocytes adhered on nanoporous CHT membranes, developing the structure of the corneum epidermal top layer, characterized by low thickness and low cell proliferation. On the microporous CHT membrane, keratinocytes formed an epidermal basal lamina, with high proliferating cells that stratified and differentiated over time, migrating along the *z* axis and forming a multilayered epidermis. This strategy represents an attractive tissue engineering approach for the creation of specific human epidermal strata for testing the effects and toxicity of drugs, cosmetics and pollutants.

## 1. Introduction

The creation of partial or complete human epidermis represents one of the major challenges in skin tissue engineering. Specific human epidermal strata are important tools for testing the effects and toxicity of drugs, cosmetics and pollutants in distinct districts of epidermis. The skin represents the primary interface between the internal and external environments, acting as barrier to protect the body from external agents and stressors, preventing unregulated water and electrolyte loss and maintaining internal homeostasis. The first physiological barrier of the skin is constituted by the epidermis, a stratified keratinized epithelium made up of migrating cells—the keratinocytes—which from the basement membrane at the dermo-epidermal junction, undergo a proliferation and differentiation process toward superficial layers, changing in composition, shape and function [1]. The engineering of physiologically relevant human skin models is an imperative need considering the international regulation requirements for skin sensitization testing, whose strategy accepts the implementation of non-animal alternatives to assess the health hazard and risks associated with potential skin sensitizers and chemicals [2]. In the European Union, since March 2013, it is no longer possible to carry out animal testing for cosmetics [3]. Hazard assessments have been progressively replaced by commercially available models of reconstructed human epidermis (RHE) such as EpiDerm™, EpiSkin^®^, SkinEthic^®^, epiCS^®^ and EPIMODEL 24. These models, validated by ECVAM (The European Centre for the Validation of Alternative Methods) for skin irritation and corrosion testing and ultimately by OECD (The Organisation for Economic Co-operation and Development) as the animal replacement of the Draize test, are widely used in phototoxicity, epidermal genotoxicity, skin sensitization and transdermal drug delivery applications [4,5,6,7,8,9]. Notwithstanding RHE models are closer to the human epidermis, their main drawnback is represented by their barrier function that does not entirely meet the requirements for drug penetration studies [10]. Moreover, they represent the entire epidermis.

Recently, specific epidermal strata were engineered by using polymeric membranes of chitosan and polycaprolactone. In particular, the different membranes, through their specific structural and physico-chemical surface properties, regulated the interaction with cells, modulating the terminal differentiation of human keratinocytes [11]. The same membranes promoted the differentiation of human dermal mesenchymal stem cells favoring the creation of human dermal–epidermal systems as full skin equivalents [12]. The use of membranes is of key interest in the development of engineered tissues. Membranes provide adaptable biomimetic microenvironments that can emulate the essential characteristics of the physiologic ones, including tissue-specific extracellular matrix interactions. Surface properties trigger specific cell responses dictating the cell fate in terms of cell growth, migration, proliferation, differentiation and functional activation [13]. Tailoring the operational parameters of membrane preparation, targeted membranes with tuned morphological, physico-chemical and mechanical properties can be developed.

In this study, we report the ability of nano- and micro-structured CHT membranes to modulate human keratinocyte stratification and differentiation. Chitosan, a copolymer derived from deacetylated chitin, is a widely used biomaterial to engineer biomimetic scaffolds for tissue engineering application, being one of the few natural polymers similar to glycosaminoglycans distributed throughout connective tissues [14]. Owing to its biodegradability, low toxicity, low immunogenicity, antimicrobial activity and haemostatic properties, chitosan has been extensively used for skin repair and regeneration [15,16]. To this purpose, nanoporous and microporous membranes of chitosan (CHT) were prepared by phase inversion technique tailoring the operational parameters in order to obtain nano- and micro-stuctured flat membranes with specific surface properties. Microporous structures with different mean pore diameters were created by adding and dissolving, in the polymeric solution, polyethylene glycol (PEG Mw 10,000 Da) as porogen, with a different CHT/PEG ratio. The developed membranes were characterized and assessed for epidermal construction by using human keratinocytes to generate an epidermal strata model. 

## 2. Materials and Methods 

### 2.1. Membrane Preparation

Chitosan (CHT) membranes were prepared in flat configuration by phase inversion technique by evaporation-induced phase separation. Nanoporous membranes were obtained by dissolving 4% (wt/v) of CHT (Mw 150,000 Da, 90–95% deacetylated, Sigma, Milan, Italy) in acetic acid solution 2% (*v*/*v*), adding polyethylene glycol (PEG, Mw 6000 Da) (Merck-Schuchardt, Hohenbrunn, Germany) at a 4:1 ratio and stirring for 2 h until complete dissolution. The polymeric solution was cast on a glass plate and molded as thin films by a handle-casting knife (Elcometer, gap set at 250 μm). The solvent evaporation was allowed in a controlled atmosphere at 20 °C, until the cast solution became a solid membrane. After the solvent evaporation, the nanoporous CHT membranes were immersed in a neutralization bath solution of 1% NaOH in order to free all the chitosan domains from the acetylation caused by the action of the solvent. The membranes were repeatedly washed with distilled water before the final drying process. 

Microstructured and microporous membranes were prepared by dissolving CHT (Mw = 150,000 Da, 90–95% deacetylated, Sigma, Milan, Italy) and PEG (Mw = 10,000 Da) used as porogen in 2% (*v*/*v*) acetic acid solution with a different (wt/v) % and ratio, respectively, as reported in Table 1. The solutions were stirred at room temperature until complete dissolution, poured on a casting apparatus, and dried at 50 °C for 8 h to allow the solvent evaporation. After drying, the membranes were immersed for 30 min in a 2% NaOH neutralization bath solution, and twice washed with deionized water to completely remove NaOH. Afterwards, membranes were kept in deionized water bath at 80 °C for 8 h in order to dissolve the PEG component and to generate the microporous structure. Finally, membranes were repeatedly washed with fresh distilled water, wiped with filter paper to remove the excess of water on their surface and allowed to dry, locked between glasses to prevent shrinkage. 

### 2.2. Membrane Characterization

After preparation, membranes were characterized in order to evaluate their morphological, physico-chemical and mechanical properties. 

The morphological and structural properties were evaluated by scanning electron microscope (SEM) (ESEM FEG QUANTA 200, FEI Company, Hillsboro, OR, USA). The membrane cross section thickness was measured by SEM images analysis and by digital micrometer Carl Mahr 40E (Esslingen, Germany).

Mean flow pore diameters and largest pores were determined by a Capillary Flow Porometer CFP 1500 AEXL (Porous Materials Inc. PMI, Ithaca, NY, USA).

Atomic force microscopy (AFM) was used to evaluate the topography and roughness of the membrane surfaces of both np CHT and mp CHT membranes in dry and wet conditions. In an AFM, a very sharp tip attached to a flexible lever interacts with the sample surface, providing information on its morphology. Data were acquired using a Multimode VIII equipped with a Nanoscope V controller (Bruker, Santa Barbara, CA, USA) in air and in a liquid environment using a fluid cell (Bruker, Santa Barbara, CA, USA). The AFM was operated in a tapping mode to avoid sample damage with a lever oscillating at a frequency of 320 kHz in air, and 7 kHz in de-ionized water (RTESPA, Bruker, Santa Barbara, CA, USA). Images were acquired on 10 × 10 µm areas, with 256 × 256 acquisition points, and scan rate of 1 Hz in air and 0.5 Hz in water. Surface roughness was estimated with respect to the mean absolute value difference (Ra) and the root mean squared difference (RMS) between the actual surface height and that of the line dividing the surface of the investigated profile into two equal areas. 

Mechanical properties of the membranes were assessed via tensile testing machine Zwich/Roell Z2.5 (Ulm, Germany). A pre-load of 0.05 MPa was applied before starting tensile tests at constant elongation rate of 4 mm/min. Real-time longitudinal deformation measurements were acquired and analyzed by testXpert® testing software. Tensile tests were carried out at 20 °C for dry and wet membranes considering the different cross section thickness. For each membrane, at least 10 double clamped strips (1 × 5 cm) were used. The tensile modulus E, evaluated from the slope of the linear portion of the stress–strain curve, the ultimate tensile strength (Rm) and the elongation at break (ε) were determined. 

Swelling tests were performed on membrane samples (1 × 1 mm) weighed and incubates in 1 mL of Dulbecco’s phosphate-buffered saline (DPBS) pH 7.4, at 37 °C, for 48 h. The swelling index (*SI*) was calculated as $SI\;\left(\%\right)=\frac{Ws-Wi}{Wi}\times 100$, where *W_i_* and *W_s_* are the sample weights before and after the incubation in DPBS, respectively. 

### 2.3. Cell Cultures

Cryopreserved human keratinocytes (HaCaT) isolated from a 62 year old male (CLS, Cell Line Service, Eppelheim, Germany) were used. Cells with a 40 population-doubling level were seeded at a cell density 6 × 10^5^ cell/cm^2^ on membranes previously UV-sterilized and conditioned with the medium constituted by DMEM with 4500 mg glucose/L (Sigma Aldrich, Milan, Italy) supplemented with 10% FCS, 2 mM Glutamine, 50 μg/mL Streptomycin and 100 U/mL Penicillin (Life Technologies, Carlsbad, CA, USA). Cells were incubated at 37 °C in a 5% CO_2_/20% O_2_ atmosphere (*v*/*v*) with 95% relative humidity.

### 2.4. Cell Morphology

The cell morphology of human keratinocytes cultured on np CHT and mp CHT membranes was investigated by Confocal Laser Scanning Microscopy (CLSM, Fluoview FV300, Olympus Italia, Segrate (MI), Italy) by proper immunostaining, after 3 and 21 days of culture. Samples were washed with PBS, fixed in paraformaldehyde, permeabilized in Triton-X100 and saturated with serum as previously described [17]. In particular, the epidermal cells were visualized for the cytoskeleton protein actin and cytokeratin CK1 and CK18. Actin was stained with Alexa Fluor^TM^ 488 phalloidin (Molecular Probes, Inc., Eugene, OR, USA) incubated for 30 min. CK1 was visualized by using a goat polyclonal antibody raised against human CK1 (Santa Cruz Biotechnology, Santa Cruz, CA, USA) and Cy^TM^5-conjugated AffiniPure donkey anti-goat IgG (Jackson ImmunoResearch Europe Ltd., Cambridge, UK). To visualize CK18, a mouse monoclonal antibody raised against human CK18 (Santa Cruz Biotechnology, Santa Cruz, CA, USA) and a Cy^TM^3-conjugated AffiniPure donkey anti-mouse IgG (Jackson ImmunoResearch Europe Ltd., Cambridge, UK) were used. Primary and secondary antibodies were incubated at room temperature for 2 and 1.5 h, respectively. Counterstaining for nuclei was performed with DAPI 0.2 mg/mL (Molecular Probes Inc., Eugene, OR, USA) incubated for 30 min. The changes of cell morphology were investigated with scan depth determination by CLSM in the *z*-scan mode (step size: 0.5 μm). Cell spreading area of human keratinocytes after 3 days of culture on the developed membranes was assessed by image analysis software (Fluoview 5.0 software, Olympus Corporation, Tokyo, Japan) on CLSM images from different areas of three samples from three separate experiments.

### 2.5. Cell Proliferation and Glucose Consumption

Cell proliferation and viability were determined by the 3-(4,5-dimethylthiazol-2-yl)-2,5- diphenyl tetrazolium bromide (MTT) assay, according to the yellow tetrazolium salt MTT reduced to purple formazan crystals by mitochondrial dehydrogenase in living cells. At different days of culture on np CHT and mp CHT membranes, and on polystyrene culture dishes (PSCD) as reference substratum, human keratinocytes were incubated with MTT 5 mg/mL for 4 h at 37 °C. The formazan crystals are then extracted by removing MTT solution and by adding in each sample 1 mL of lysis buffer constituted of 10% sodium dodecyl sulphate (SDS), 0.6% acetic acid in DMSO and mixing for 30 min at 37 °C. The formazan product was quantified by spectrophotometry at 570 nm wavelength.

Glucose consumption was detected at different days of culture of the human keratinocytes on np CHT and mp CHT membranes, and on polystyrene culture dishes (PSCD) as reference substratum, by Accu-Chek Active assay (Roche Diagnostics, Monza, Italy).

### 2.6. Statistical Analysis

Statistical analysis was performed using the ANOVA followed by Bonferroni’s *t*-test (statistical significance *p* < 0.05), and Student’s *t*-test (statistical significance *p* < 0.01 and *p* < 0.05).

## 3. Results and Discussion

### 3.1. Membrane Properties

The morphological, physico-chemical and mechanical properties of the developed nano- and micro-porous CHT membranes were assessed before their use for epidermal construction. 

**Figure 1:**
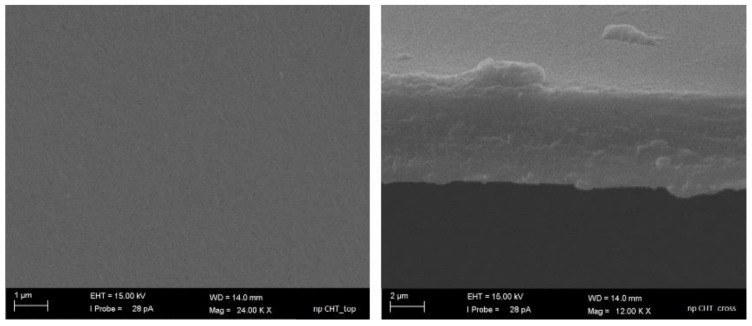
SEM pictures of top surface (**a**) and cross-section (**b**) of nanoporous chitosan membrane.

As evidenced by SEM images, the nanoporous CHT membrane revealed a homogeneous and smooth surface (Figure 1a) and a cross section with a thickness of 5.5 ± 0.9 µm (Figure 1b). The capillary flow porometer analysis demonstrated the presence of open nanopores with mean flow diameter of 26 nm. 

Microporous membranes were prepared by dissolving CHT and PEG Mw 10,000 Da as porogen in a different (wt/v) % and ratio in the casting solution, as reported in Table 1. After the evaporation-induced phase separation process, the porogen was dissolved in a water bath at 80 °C. This treatment allowed the selective removal of PEG while keeping the polymer structure unaltered, since chitosan is stable at this temperature as known from thermogravimetric curve of the polymer (with 94% degree of deacetylation) [18,19]. After extensive washing and the drying process, microstructured membranes with a thickness of 22.6 ± 0.1 µm were obtained. 

**Figure 2:**
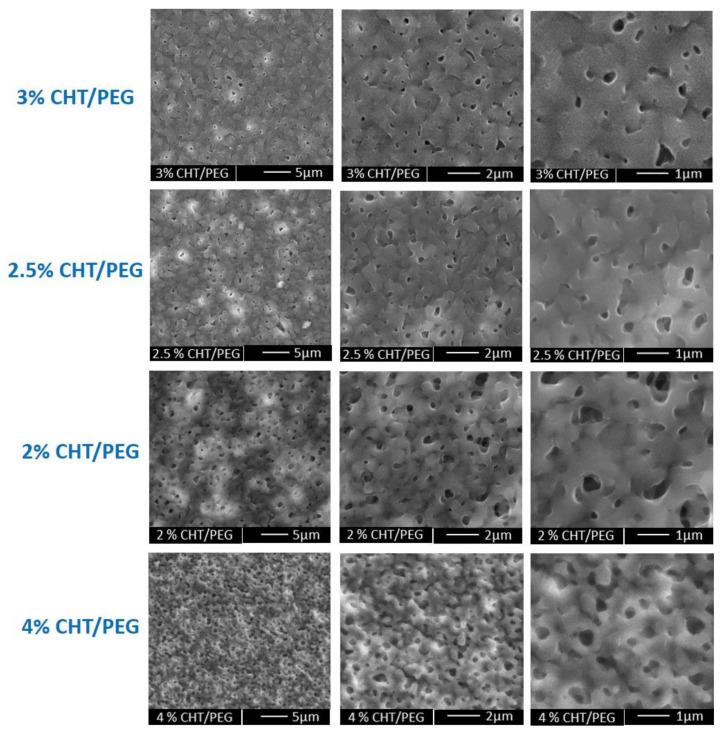
SEM pictures at different magnification of microporous membranes obtained by casting polymeric solution with different (wt/v) % of CHT/PEG, as reported in Table 1.

The SEM micrographs at different magnification show the surface morphology of the membranes prepared from a different CHT/PEG ratio (Figure 2). It is noteworthy that the greater CHT/PEG ratio in the casting solution induced the formation of lower pore size in the membranes since, typically, a higher concentration of polymer composition enhances the precipitation, suppressing the pore size growth. Additionally, the porogen dissolution process plays a role in the pore formation as demonstrated by the dense structure displayed by membranes that did not undergo to the porogen removal after the polymer casting (Figure S1, Supplementary Materials). The dimension of open pores in all the developed microstructured CHT membranes were assessed by capillary flow porometer analysis. As reported in Table 1, the results confirmed that the open pore size increased with decreasing of the CHT/PEG ratio. Although open pores are present in all membranes, mean flow pore diameters of 0.131 ± 0.072 µm and largest pores diameters of 0.849 ± 0.772 µm were measured only in the membrane obtained by the 2% CHT/PEG casting solution. This membrane, named as microporous chitosan membrane (mp CHT), was selected for a deep characterization and utilized for epidermal construction in comparison with the nanoporous membrane (np CHT). Increasing the (wt/v) % of both the polymer and porogen (4% CHT/PEG), the pores distribution seems to increase but the mean flow pore diameters and the largest pores decreased in comparison to the membranes with the same CHT/PEG ratio (2% CHT/PEG) (Figure 2 and Table 1). Since the polymer is the component that forms the membrane matrix, higher polymer concentration in the casting solution decreases the final porosity. The increase in polymer concentration results in an increase in the viscosity of the solution that slowed down the out diffusion of the solvent, and thereby decreased the porosity of the cast membrane.

**Figure 3:**
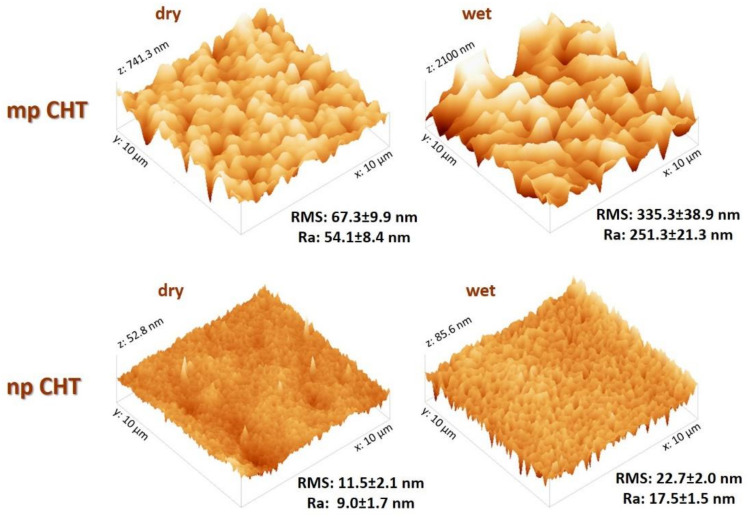
AFM images of 10 × 10 µm microporous chitosan (mp CHT) and nanoporous chitosan (np CHT) membrane surfaces in dry and wet conditions, and relative roughness values in terms of Ra (average absolute distance from average flat surface) and RMS (root mean square from average flat surface).

Further investigations were performed by AFM analysis to evaluate the surface topography and roughness of the np CHT and mp CHT membranes in dry and wet conditions (Figure 3). The surface roughness is a critically important parameter in cell material interaction that triggers specific cell response, playing a crucial role in epidermal differentiation [11]. Consistently with SEM results, the AFM analysis highlighted the uniform smooth surface of np CHT membrane, which exhibited roughness values of 11.5 ± 2.1 and 9.0 ± 1.7 nm of RMS and Ra, respectively. The presence of micropores on the surface of the mp CHT membrane significantly enhanced the roughness in comparison to the np CHT membranes, reaching values of 67.3 ± 9.9 and 54.1 ± 1.7 nm of RMS and Ra, respectively. In wet conditions, the surface topography of both the np CHT and mp CHT membranes changed, as demonstrated by the Ra parameter which increased by more than 90% for np CHT membrane and more than 360% for the mp CHT membrane in comparison with the dry membranes. Relevant roughness parameters were measured for the wet microporous membrane which displays values of 335.3 ± 38.9 and 251.3 ± 21.3 nm of RMS and Ra, respectively.

Both the np and mp CHT membranes incorporate water when wet due to the hydrophilic character of chitosan. The mechanism is determined mainly by ionic interactions (electrostatic repulsion) between chitosan chains and by solvent diffusion since the amino groups on chitosan tend to remain protonated at physiologic pH 7.4. A sustained swelling index was displayed by the mp CHT membrane which, after 48 h of incubation at 37 °C in physiologic saline DPBS buffer solution, acquires an aqueous volume of 345 ± 66%, in agreement with the % roughness increase. The microporous structure of mp CHT confers to the membrane a high-water adsorbent capacity with significantly higher values of swelling than that measured for the np CHT (211 ± 48%). The water incorporation influenced the membranes topographical and mechanical properties, as highlighted by AFM and mechanical tests results.

**Figure 4:**
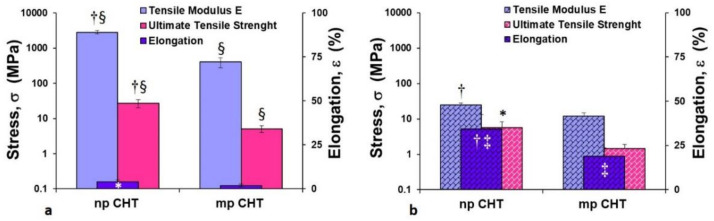
Mechanical properties of nanoporous (np CHT) and microporous (mp CHT) chitosan membranes in dry (**a**) and wet (**b**) conditions. The values are the means of 10 measurements per sample ± SD. Data are statistically significant according to Student’s t-test: (†) *p* < 0.01 and (*) *p* < 0.05, vs. mp CHT, in the same dry or wet conditions; (§) *p* < 0.01 vs. wet condition on the same substratum; (‡) *p* < 0.01 vs. dry condition on the same substratum.

Considering that the mechanical characteristics of the surrounding microenvironment strongly affects morpho-functional behavior, dynamics and three-dimensional rearrangement of the cells, the mechanical properties of the membranes in both dry and wet conditions were further evaluated (Figure 4).

The microporous structure of the mp CHT membrane was responsible for lower values of tensile modulus E (402.5 ± 124 MPa) and ultimate tensile strength (5.1 ± 1.1 MPa) in dry conditions compared to the values exhibited by the np CHT membrane, which had a stiffer character. Under wet conditions for both the np CHT and mp CHT membranes, the tensile modulus E and ultimate tensile strength drastically decreased (about 100-fold), and simultaneously the percentages of elongation ε at break increased (about 10-fold) (Figure 4b). The incorporation of water molecules re-established new hydrogen bonds between the polymer chains, which become able to slide over one other under stress. Increments of applied forces corresponded to lower resistance to deformation (which were translated in decreased values of tensile modulus E of 24.4 ± 3.5 and 12.1 ± 2.8 MPa, and UTS of 5.7 ± 2.6 and 1.5 ± 0.4 MPa, for the np CHT and mp CHT membranes, respectively), and greater increase in elongation (34.4% and 18.8% for the np CHT and mp CHT membranes, respectively). The mechanical properties variation due to the water incorporation in polymeric chains was also observed in polycaprolactone (PCL) and CHT/PCL nanoporous and double porous membranes, as well as in HF PCL membranes [12,20,21]. 

### 3.2. Epidermal Membrane Sytems

**Figure 5:**
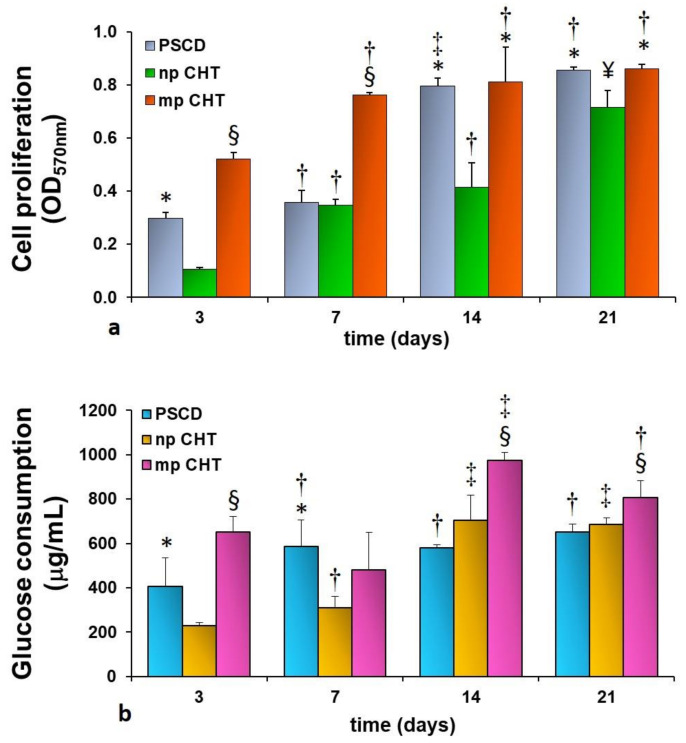
Cell proliferation (**a**) and glucose consumption (**b**) of human keratinocytes at different days of culture on nanoporous chitosan (np CHT) and microporous chitosan (mp CHT) membranes, and on polystyrene culture dishes (PSCD) as reference substratum. The values are the mean ± standard deviations of three experiments per configurations. Data are statistically significant according to ANOVA followed by Bonferroni’s *t*-test (*p* < 0.05): (*) vs. np CHT, (§) vs. np CHT and PSCD, at the same day of culture; (†) vs. day 3, (‡) vs. days 3 and 7, (¥) vs. days 3, 7 and 14 of culture, on the same substratum.

Epidermal membrane systems were realized by culturing human keratinocytes on the developed np CHT and mp CHT membranes. Cell proliferation was investigated for up to 21 days of culture. Human keratinocytes on polystyrene culture dishes (PSCD) were used as reference substratum. As reported in Figure 5a, a sustained proliferation was displayed by the cells cultured on the mp CHT membrane, with values that increased with time, and reaching the maximum value (OD = 0.861 ± 0.042) at day 21. The growth rate on the membrane was significantly higher than that exhibited by the cells cultured on np CHT, for the whole culture time, and by the cells on the reference substratum, in the first week of culture. Differently, human keratinocytes cultured on np CHT membrane undergo a moderate proliferation with significantly lower values, especially in the early days of culture. The functional feature of the cells was assessed by monitoring the glucose consumption, the glucose being an important nutrient and the primary source of energy in sustaining cell growth and viability. Consistently with cell growth, the cellular glucose uptake followed the same trend (Figure 5b) as a result of the enhanced metabolic request for proliferating keratinocytes. A peak of glucose consumption of 975 ± 35 µg/mL was reached at day 14 by human keratinocytes cultured on the mp CHT membrane.

**Figure 6:**
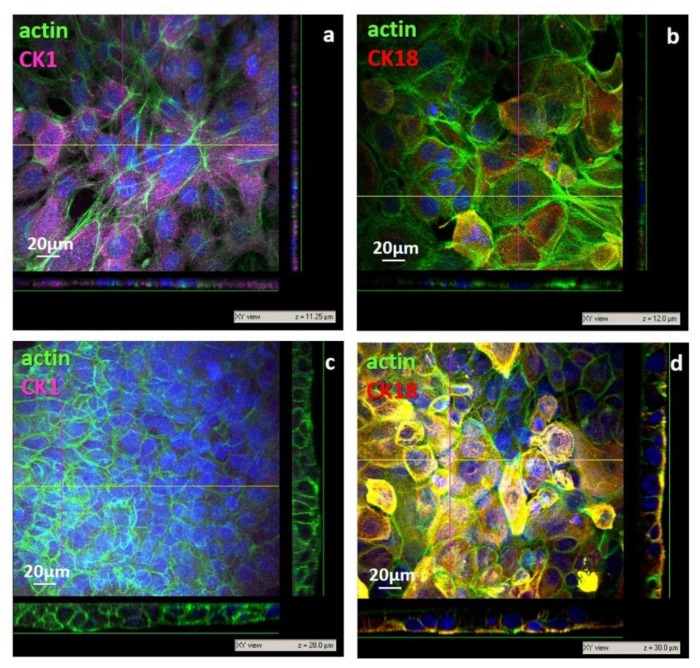
CLSM images of primary human keratinocytes after 3 days of culture on nanoporous chiosan (np CHT) (**a**,**b**), and microporous chitosan (mp CHT) (**c**,**d**) membranes: *xy* views and relative *xz* and *yz* projections along *z* axis, with relative thickness values (µm). Cells were visualized for actin (green), CK1 (magenta), CK18 (red) and nuclei (blue).

The cell morphology, investigated by confocal laser scanning microscopy, highlighted a remarkable difference in the behavior of human keratinocytes after 3 days of culture on the developed membranes. On the nanoporous membrane, cells adhered exhibiting a flattened shape with a high spreading degree of 1157 ± 573 µm^2^/cell, ranging from 3447 to 403 µm^2^ (Figure 6a,b). The cell flattening was also demonstrated by the *xz* and *yz* projection of the confocal images that displayed, on the np CHT membrane, epidermal membrane systems with thickness of 10–12 µm (Figure 6a,b). The moderate surface roughness of the np CHT membrane enhanced the cell flattening that triggered the keratinocytes differentiation to the corneum layer, the outermost layer of epidermis, in which cells are extremely flattened, not proliferating and express specific pattern of cytokeratins. In particular, CK1, a marker of the suprabasal level of epidermis, after only 3 days was strongly expressed in human keratinocytes cultured on np CHT. On the contrary, CK18, assessed as a marker of cell proliferation, was found weakly expressed on the np CHT membrane (Figure 6b), accordingly with the low cell proliferation and energetic metabolism data (Figure 5). This membrane seems to induce the terminal keratinocytes differentiation in the outermost skin layer. 

After 3 days of culture, small and densely packed cells were found in adhesion to the microporous surface of the mp CHT membrane, with a spreading degree of 330 ± 107 µm^2^ and an elongated columnar shape, as shown in the *xz* and *yz* projection of confocal images (Figure 6c,d), like the cells of the basal lamina of epidermis. Cells with more cuboidal shape and an enlarged surface of 765 ± 230 µm^2^ were found in the upper level. The multistrata growth and disposition, with flattening of cells moving from the bottom to the top levels, was observed in confocal acquisition along the *z* axis, as illustrated in the *xz* and *yz* projection of CLSM image, reaching thickness of 28–30 µm (Figure 6c,d), which is peculiar of viable epidermis [1]. It should be noted that unlike the human keratinocytes cultured on the nanoporous membrane, cells on the mp CHT membrane after 3 days of culture exhibited a remarkable expression of CK18 (Figure 6d) as a result of their sustained growth activity, also corroborated by the cell proliferation and glucose consumption (Figure 5). Moreover, the quite missing CK1 expression (Figure 6c) evidenced that cells did not reach their terminal differentiation belonging to keratinocytes of the suprabasal corneum layer. 

**Figure 7:**
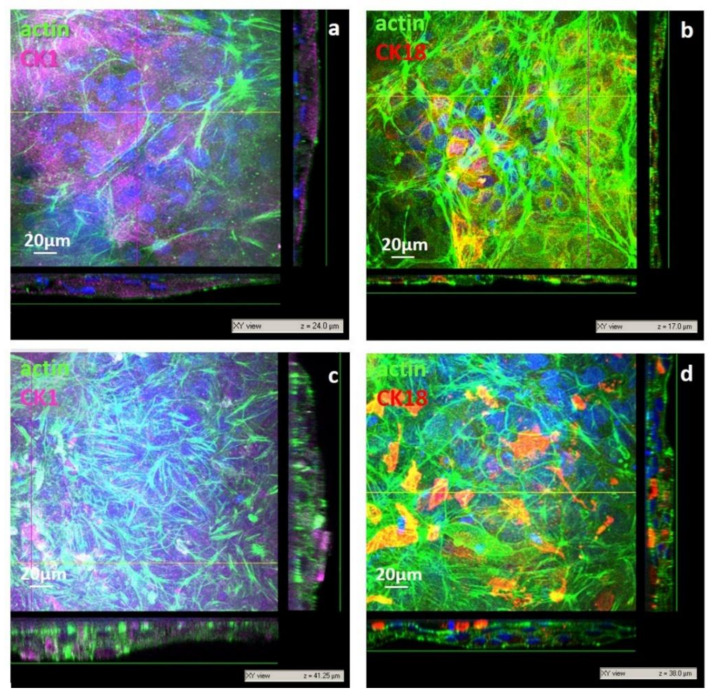
CLSM images of primary human keratinocytes after 21 days of culture on nanoporous chitosan (np CHT) (**a**,**b**) and microporous chitosan (mp CHT) (**c**,**d**) membranes: *xy* views and relative *xz* and *yz* projection along *z* axis. Cells were visualized for actin (green), CK1 (magenta), CK18 (red) and nuclei (blue).

After 21 days on the np CHT membranes, cells grew, increasing their cell density and stratification with the formation of thicker sections (17–24 µm, Figure 7a,b). The expression of CK1 became highly significant after 21 days in the cells cultured on the mp CHT membrane, where keratinocytes grew as compact multilayers with thickness of 38–42 µm (Figure 7c,d) as an indication of their terminal differentiation. Cells in the lower strata, in direct contact with the membrane, exhibited actin stress fibers as a result of their anchoring bridges to the microporous structure of membrane surface (Figure S2, Supplementary Materials). Scaling to the upper layers, the human keratinocytes were found more cuboidal, and then more enlarged and flattened, reaching a spreading area of 1132 ± 249 µm^2^ in the top layers. The strong expression of CK1 found in the upper layers supported the cell capability to stratify and undergo terminal differentiation. The microporous structure and surface roughness of membranes in aqueous medium guided morpho-functional changes that are typical of keratinocytes belonging to the basal lamina, which in vivo are responsible of the stratification and migratory differentiation of keratinocytes, forming the viable and self-renewing epidermis. 

A similar behavior was found on nanoporous polycaprolactone membranes, which exhibited topographical peculiar surface microstructures and elastic properties that were maintained more stable under wet conditions (tensile modulus E of 443 and 344 MPa, in dry and wet conditions, respectively) [11]. Tensile tests on straight collagen fibers of in vivo dermis reported tensile modulus E ranging from 100 to 1000 MPa [22]. The mechanical properties of both np and the mp CHT membranes drastically changed after water incorporation. Interestingly, it has been reported that in-plane tensile studies of human stratum corneum exhibited a sustained mechanical variation, with moduli decreasing from 1000 to 5 MPa with increasing hydration [23]. Similar findings were displayed by measuring the elastic and viscoelastic properties of stratum corneum through nanoindentation techniques, with values of 100 and 10 MPa, for dry and wet stratum corneum [24]. Other authors reported slight lower values of Young’s moduli for viable epidermis with respect to the stratum corneum [25,26]. The broad range of values is likely caused by the differences in testing apparatus and protocols, variety among species and body sites, and heterogeneity of the materials. Wet mp CHT membranes, which since the early days of culture sustained the differentiation of viable epidermis, exhibited lower Young’s moduli in comparison to the wet np CHT, which promoted the differentiation of outer corneum epidermal layer. Moreover, wet mp CHT membranes exhibited Young’s moduli values of 12 MPa, similar to the values of 13.5 MPa measured by Crichton et al., for freshly isolated dermis [26].

## 4. Conclusions

This study highlights how it is possible to stimulate the formation of epidermal strata by creating membranes with tailored properties. The design of membranes with specific functionalities and structural features, through the modulation of preparation process parameters, represents a challenging strategy to stimulate the interactions with cells and their morpho-functional behavior.

The surface topography of nano- and micro-structured membranes strongly affected the cell fate of human keratinocytes, guiding their proliferation and differentiation. The different CHT membranes induced specific cellular responses. In particular, nanoporous CHT membrane—with nanopores of 26 nm, moderate roughness and tensile modulus E of 24.4 ± 3.5 MPa under wet conditions—after 3 days drove the terminal keratinocytes differentiation, developing the structure of the corneum epidermal top layer, characterized by low thickness, low cell proliferation and high expression of CK1. The microporous CHT membrane—with open pore diameter of 0.131 µm, enhanced roughness and tensile modulus E of 12.1 ± 2.8 MPa under wet conditions—induced the formation of an epidermal basal lamina, with high proliferating cells that stratified and differentiated over time, migrating along the *z* axis and forming a multilayered and viable epidermis.

The above-described achievements towards the creation of membranes with specific cues represent a contribution to the comprehensive understanding of cell-material interactions that have become increasingly prominent in the field of tissue engineering and regenerative medicine. This strategy represents an attractive tissue engineering approach for the creation of specific human epidermal strata for testing the effects and toxicity of drugs, cosmetics and pollutants.

## Figures and Tables

**Table 1:** Composition of the casting solutions in terms of chitosan (CHT) (wt/v) % and CHT/polyethylene glycol (PEG, Mw 10,000 Da) ratio, for the development of different microstructured CHT membranes, and corresponding values of the membrane open pores diameters detected by Capillary Flow Porometer.

Casting Solution	CHT (wt/v) %	CHT/PEG Ratio	Mean Flow Pore Diameter (µm)	Largest Detected Pore Diameter (µm)
3% CHT/PEG	2	2/1	0.033 ± 0.008	0.419 ± 0.024
2.5% CHT/PEG	1.5	1.5/1	0.054 ± 0.037	0.447 ± 0.094
2% CHT/PEG	1	1/1	0.131 ± 0.072	0.849 ± 0.772
4% CHT/PEG	2	1/1	0.042 ± 0.081	0.420 ± 0.091

## Supplementary Materials

The following are available online at https://www.mdpi.com/article/10.3390/membranes11060394/s1. Figure S1: SEM images at different magnification of membranes obtained by 2.5% CHT/PEG without the porogen dissolution process, Figure S2: Z-stack images of human keratinocytes after 21 days of culture on microporous chitosan (mp CHT) membrane from the bottom (a) to the uppermost (i) layers. Images were collected at 0.5 μm intervals. Cells were stained for actin (green), cytokeratin CK1 (magenta) and counterstained for nuclei (blu). Scale bar 20 μm.
