# Supplementary material for: Nano- and Micro-Porous Chitosan Membranes for Human Epidermal Stratification and Differentiation
Source: Membranes (Basel). 2021 May 27;11(6):394. doi: 10.3390/membranes11060394 (PMC8227018; doi:10.3390/membranes11060394)
Supplement: Supplementary file 1 [file membranes-11-00394-s001.zip › membranes-1214435-supplementary.pdf]

Supplementary Data

Nano- and Micro-Porous Chitosan Membranes for Human Epidermal Stratification and Differentiation

Simona Salerno [1,*], Maria Penelope De Santo [2], Enrico Drioli [1,3] and Loredana De Bartolo [1,*]

1 Institute on Membrane Technology, National Research Council of Italy (CNR-ITM), via P. Bucci, cubo 17/C, 87036, Rende (CS), Italy; e.drioli@itm.cnr.it (E.D.)
2 Department of Physics and CNR-Nanotec, University of Calabria, via P. Bucci cubo 31/C, 87036, Rende (CS), Italy maria.desanto@fis.unical.it (M.P.D.S.)
3 College of Chemical Engineering, Nanjing Tech University, Xinmofan Road, Nanjing 210009, China

* Correspondence: s.salerno@itm.cnr.it, Tel.: +39 0984 492034 (S.S.); l.debartolo@itm.cnr.it Tel.: +39 0984 492036 (L.D.B.).

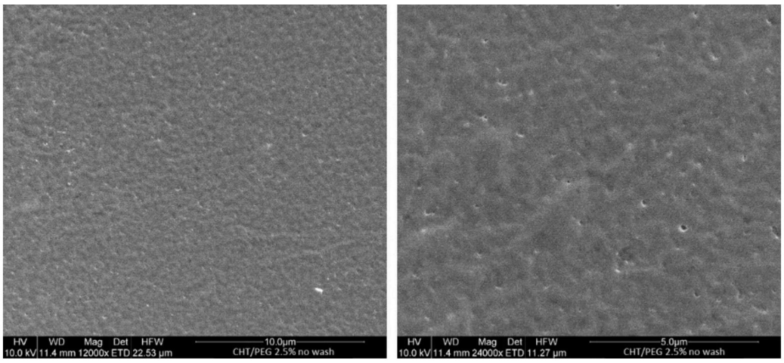

Figure S 1. SEM images at different magnification of membranes obtained by 2.5% CHT/PEG without the porogen dissolution process.

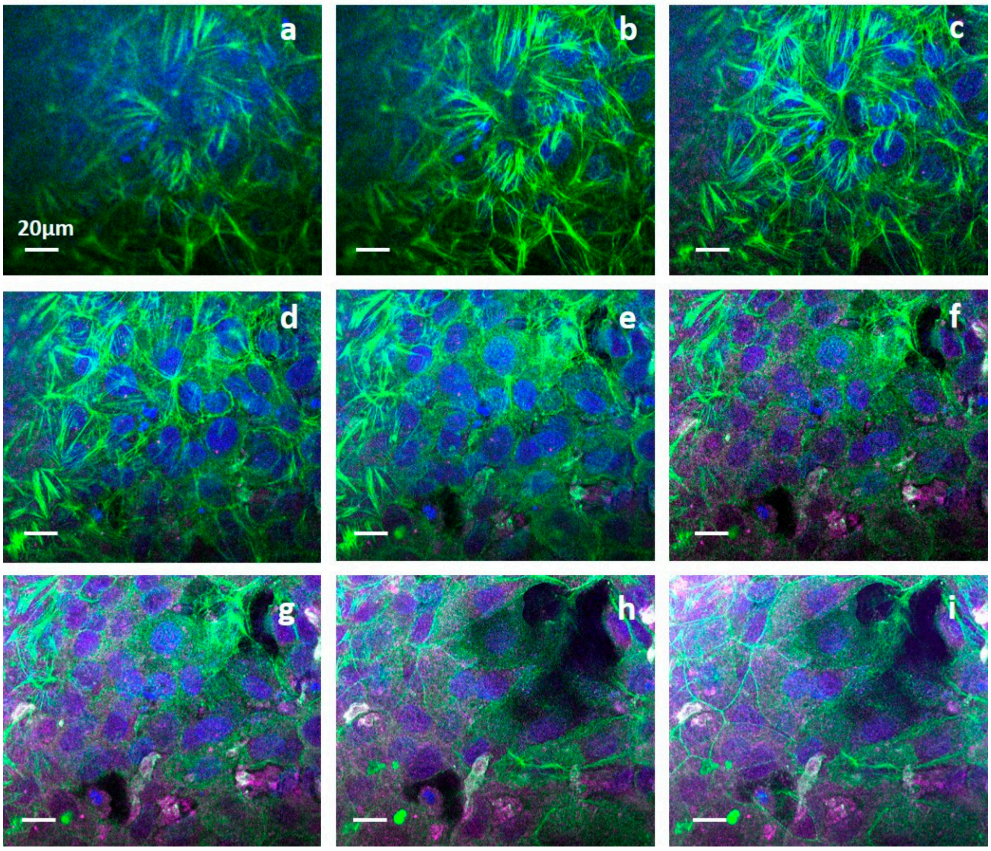

Figure S 2. *Z*-stack images of human keratinocytes after 21 days of culture on microporous chitosan (mp CHT) membrane from the bottom (a) to the uppermost (i) layers. Images were collected at 0.5 µm intervals. Cells were stained for actin (green), cytokeratin CK1 (magenta) and counterstained for nuclei (blu). Scale bar 20 µm.
